# Foot-related diabetes complications: care pathways, patient profiles and costs

**DOI:** 10.1186/s12913-022-07853-2

**Published:** 2022-04-26

**Authors:** Olli Kurkela, Jaakko Nevalainen, Martti Arffman, Jorma Lahtela, Leena Forma

**Affiliations:** 1 Faculty of Social Sciences, Tampere University, P.O Box 100, 30014 Tampere, Finland; 2 Welfare State Research and Reform Unit, Finnish Institute for Health and Welfare (THL), P.O. Box 30, 00271 Helsinki, Finland; 3 Tampere University Hospital, Teiskontie 35, P.O. Box 2000, 33520 Tampere, Finland; 4 Faculty of Social Sciences, University of Helsinki, PO 54, 00014 Helsinki, Finland; 5 Laurea University of Applied Sciences, Ratatie 22, 01300 Vantaa, Finland

**Keywords:** Diabetes complications, Diabetic foot, Care pathways, Healthcare costs, Sequence analysis

## Abstract

**Background:**

Foot-related diabetes complications reduce individual well-being, increase mortality and results in increased healthcare costs. Despite their notable stress on health services, studies examining the foot complication care pathways, especially from the viewpoint of health services, are limited. We aimed to identify the most typical care pathways following an initial foot-related diabetes complication, to characterize the patients on each pathway and calculate the related healthcare costs.

**Methods:**

The identification of pathways was based on population-wide register-based data including all persons diagnosed with diabetes in Finland from 1964 to 2017. For each patient, initial foot-related complication from 2011–2016 was identified using the ICD-10 codes and related healthcare episodes were followed for two years until the end of 2017 or death. A sequence analysis was conducted on care episodes resulting in groups of typical care pathways, as well as their patient profiles. The costs of pathways resulting from the care episodes were calculated based on the data and the reported national unit costs and analyzed using linear models.

**Results:**

We identified six groups of typical pathways each comprising mainly single type of care episodes. Three of the groups comprised over 10 000 patients while the remaining groups ranged from a few hundred to a few thousand. Majority of pathways consisted only single care episode. However, among the rest of the care pathways variability in length of care pathways was observed between and within group of pathways. On average, the patients were over 65 years of age and were diagnosed with diabetes for over a decade. The pathways resulted in an annual cost of EUR 13 million. The mean costs were nearly 20-fold higher in the group with the highest costs (EUR 11 917) compared to the group with the lowest costs (EUR 609).

**Conclusions:**

We identified groups of typical care pathways for diabetic foot and discovered notable heterogeneity in the resource use within the groups. This information is valuable in guiding the development of diabetes care to meet the growing need. Nevertheless, reasons underlying the observed heterogeneity requires further examination. Since foot complications are largely preventable, substantial savings could be achieved using cost-effective technologies and more efficient organization of care.

**Supplementary Information:**

The online version contains supplementary material available at 10.1186/s12913-022-07853-2.

## Background

Foot complications develop as a result of complex micro- and macrovascular dysfunctionalities [1]. Major precursors for foot ulcers are peripheral vascular disease, peripheral neuropathy and other microvascular complications. Aging and longer duration of diabetes increase the risk of foot complications, particularly foot ulcers, and the risk is observed to be higher among males compared to females and among patients with type 2 diabetes compared to type 1 [1, 2]. The risk of mortality within five years is reported to be 2.5-fold higher among patients with an initial ulcer compared to patients without ulcer. However, other comorbid micro- and macrovascular diabetes complications notably contribute to the total risk [1, 3].

In 2017, the age-standardized prevalence of type 1 and type 2 diabetes in Finnish population was 96 and 725 per 10 000 person years, respectively [4]. The age-standardized incidence and prevalence of diabetic foot was 111 and 989 per 10 000 people with diabetes in 2015–2017. Most common conditions were peripheral artery disease (592 per 10 000 people with diabetes), ulcers (527), amputations (101) and Charcot foot (42) [5].

The economic burden associated with diabetic foot complications is substantial. The American Diabetes Association (ADA) has estimated that in the USA, one third of diabetes expenditure is due to foot complications [6] and in the United Kingdom, approximately 0.6% of the total National Health Service budget is allocated to the treatment of foot complications [7]. The costs of care vary substantially depending on the severity of the condition and how health services are organized [8, 9].

In Finland, public health care is available to all citizens and is partially complemented by private sector. The responsibility of care is shared between primary care, specialized care units (local and university hospitals), private services and occupational health care [10]. The prevention and management of foot complications is a multidisciplinary effort that is directed by national guidelines and involve multiple specialties and healthcare sectors [11, 12]. To date, the costs of foot complications in Finland have not been studied.

Typical causal pathways describing the development of foot complications have been well documented [1]. However, studies from a health service perspective are limited, despite the substantial economic burden related to foot complications [13]. Previous studies have been prospective studies that followed a small number of selected patients [14] or reported annual resource use or costs of foot complication treatment, with primary emphasis on diabetic ulcers [7, 9, 15].

We addressed this knowledge gap by representing the individual care pathways for diabetic foot in terms of actualized care episodes. The care pathways were then grouped according to their similarity to identify typical care pathways of diabetic foot. More specifically, we aimed 1) to define the most typical care pathways for treating the initial diagnosed foot complication, 2) to characterize the patients on each care pathway and 3) to calculate resource use and costs related to each care pathway. To achieve this, we adopted a data-driven approach based on the mining of register data of over 50 000 patients. This study provides a coherent picture of the multidisciplinary care of diabetes foot complications and associated costs. A more coherent picture could inform planning and organization of health services related to the prevention and management of diabetes foot complications.

## Methods

### Register data

The data were based on the Diabetes in Finland (FinDM) cohort, which comprises all people diagnosed with diabetes in Finland from 1964–2017 [4]. The data have been gathered from several population-wide administrative healthcare registers and have been linked using personal identity codes [16]. The data include detailed information on all care episodes in public primary and specialized health care settings, thereby covering most diabetic foot care provided in Finland. By including information on entry and departure, main and auxiliary diagnoses, operational codes, as well as cost information using the diagnosis related group (DRG) system, the data enabled the detailed day-to-day investigation of care episodes and the identification of care pathways. A full description of data collection has been previously reported [4].

### Study population

From the entire FinDM cohort (*n =* 887 210), we identified patients who had their initial foot-related care episode in 2011–2016 (*n =* 63 900, Supplementary Fig. 1). By restricting our time frame to this period, we were also able to include data on primary outpatient care. To ensure that this was the first complication, a three-year washout period with no complications before the onset of diabetes was applied. The care episodes mainly occurred during the first two years after the initial foot-related care episode, and we focused on that time period.

### Care pathways

The starting point of a care pathway was defined as the entry date of the initial foot-related care episode. Each pathway only included care episodes and procedures related to diabetic foot detected using ICD-10 and procedure codes (Table 1). Thus, this study focused on care related to diabetic foot, excluding care for other conditions. Each specialty that contributes for treatment of diabetic foot uses discipline-specific ICD-10 codes, and the codes were grouped accordingly (dermal, infection, neuropathy, orthopedic, vascular). The care pathways were followed for two years (*n =* 41 635) or until the event of earlier death (*n =* 6 692).

**Table 1:** ICD-10 codes used to detect foot complications and comorbid late-stage complications

Group	Description	ICD-10 codes
Dermal	ulcers, corns and callosities, epidermal thickenings, gangrene, ingrowing nails	L97, L84, L85, L89, R02, S91.1, S91.2, S91.3, S91.7, L60.0
Infection	cellulitis and acute lymphangitis, dermaphytosis, and viral warts	L03.0, L03.1, B35.1, B35.3, B07.9
Neuropathy	polyneuropathy, other diseases of nervous system	G63.2, G99.0
Orthopedic	arthopathies, osteomyelitis, acquired deformities of limbs, fingers and toes	M14.6, M86, M20.1, M20.2, M20.3, M20.4, M20.5, M20.6, M21.4, M21.5
Vascular	peripheral angiopathy, atherosclerosis	I79.2, I70.9
**Comorbid late-stage complications**		
Coronary artery disease		I20, I21, I22, I23, I24, I25, R96, R98
Diabetic retinopathy		H34, H54, E103., E11.3, E12.3, E13.3, E14.3, H28.0, H36.0, H40.5, H42.0, H40.5, H42.0, H43.1, H45.0, CKC12, CKD05, CKD60, CKD65
Nephropathy		N18, Z49, Z94, N08.3
Neuropathy		E10.4, E11.4, E12.4, E13.4, E14.4, G57.1, G57.3, G57.5, G59.0, G63.2, G73.0, G99.0, I95.1, N48.4

### Costs of care pathways

We adapted the healthcare perspective when calculating the costs of care pathways. The costs resulted from outpatient and inpatient care episodes in both public primary and public and private specialized health care settings.

The calculation of costs was based on the expenses information in healthcare registers and, when not available, the reported national unit costs from 2011 by the Finnish Institute for Health and Welfare [17]. The report includes DRG-specific costs from 2011 for care episodes in specialized health care and the national mean costs of visits and inpatient days for primary and specialized care. Each approach enables calculation of aggregate costs of each care episode, including costs of inspection, procedures and medication administered during the care episodes.

The cost of each primary outpatient care episode was calculated using the national mean cost of a primary outpatient care episode specific to a healthcare professional’s role and type of contact. The cost of each primary inpatient care episode was calculated by multiplying the national mean cost of a primary inpatient day based on the length of stay (less or more than 90 days) by the length of stay [17].

The cost of each specialized care episode was primarily calculated using the register information on care episode-specific cost and, secondarily, using DRG-specific cost. If these were not available (~ 5% of pathways), we used national average cost according to hospital level and specialty of care [17]. The costs were converted into 2017 price level using the price index for public expenditure provided by Statistics Finland [18] and into US dollars using an exchange rate of 1.1993 (31 December 2017) [19].

### Statistical analyses

Our analysis started by identifying groups of care pathways using sequence analysis. This was followed by an investigation of patient profiles in the groups and their average and total costs. The heterogeneity of each group of pathways was further decomposed by tree diagrams.

Event sequences were defined as follows: time origin was set as the entry date of the initial foot-related care episode, and one week as the unit of time. The states in a sequence represented either a care episode, absence of care episodes, multiple care episodes or deceased state. We grouped the care episode diagnoses into five groups: dermal, infection, neuropathy, orthopedic and vascular (Table 1), each of which represented a care episode. Each state was then assigned one of eight labels: one of the diagnosis group labels, “no care episodes”, “multiple care episodes” or “deceased”. A total of 104 states – one for each week – were defined for each person.

Hierarchical clustering algorithm (Ward method) was performed to obtain groups of similar sequences (i.e. groups of pathways) based on the individual state sequences over time. Similarity between sequences was defined as the sum of “costs” assigned to the insertion, deletion and substitution of states necessary to transform one sequence into another (optimal matching algorithm). We assigned a “cost” of 1 for insertions and deletions, and transition rate specific costs for substitution (“cost” > 1). The substitution cost of the abundant “no care episode” state for any other state was set to one. Patients who died during the first two years were not included in the cluster analysis and were treated as a separate group of pathways.

Due to computational restrictions, we ran the cluster analysis in two randomly sampled subsets. The number of groups of pathways was selected based on the height of the consecutive steps in a dendrogram (scree plots). From both subsets, we distinguished eight groups of very similar pathways, which were manually merged.

We characterized patient profile of each group by their background variables: age, duration of diabetes at the start of the follow-up, type of diabetes, sex and late-stage complications (coronary artery disease, diabetic retinopathy, nephropathy, neuropathy [1]. A patient was considered as having been treated for a specific late-stage complication if at least one of the care episodes in the patient’s pathway had a late-stage complication diagnosis entry (ICD-10 codes in Table 1).

We analyzed the costs of groups of pathways using linear models. In the first model, we estimated the mean costs for each group of pathways to illustrate crude differences. In the second model, the means and differences were adjusted for sex, age (linear and quadratic terms), diabetes type, duration of diabetes, year of the initiation of the pathway and whether or not patient was diagnosed with comorbid late-stage complications (retinopathy, nephropathy and coronary artery disease). The Deceased group was excluded from the analysis. The heterogeneity of each group of pathways was further visualized using tree diagrams. The most frequent pathways in each group were manually grouped based on the type and number of care episodes, represented by a tree branch.

All analyses were carried out using R statistical software (version 3.6.2) and the TraMiner package [20–22].

## Results

### Typical care pathways

**Fig. 1:**
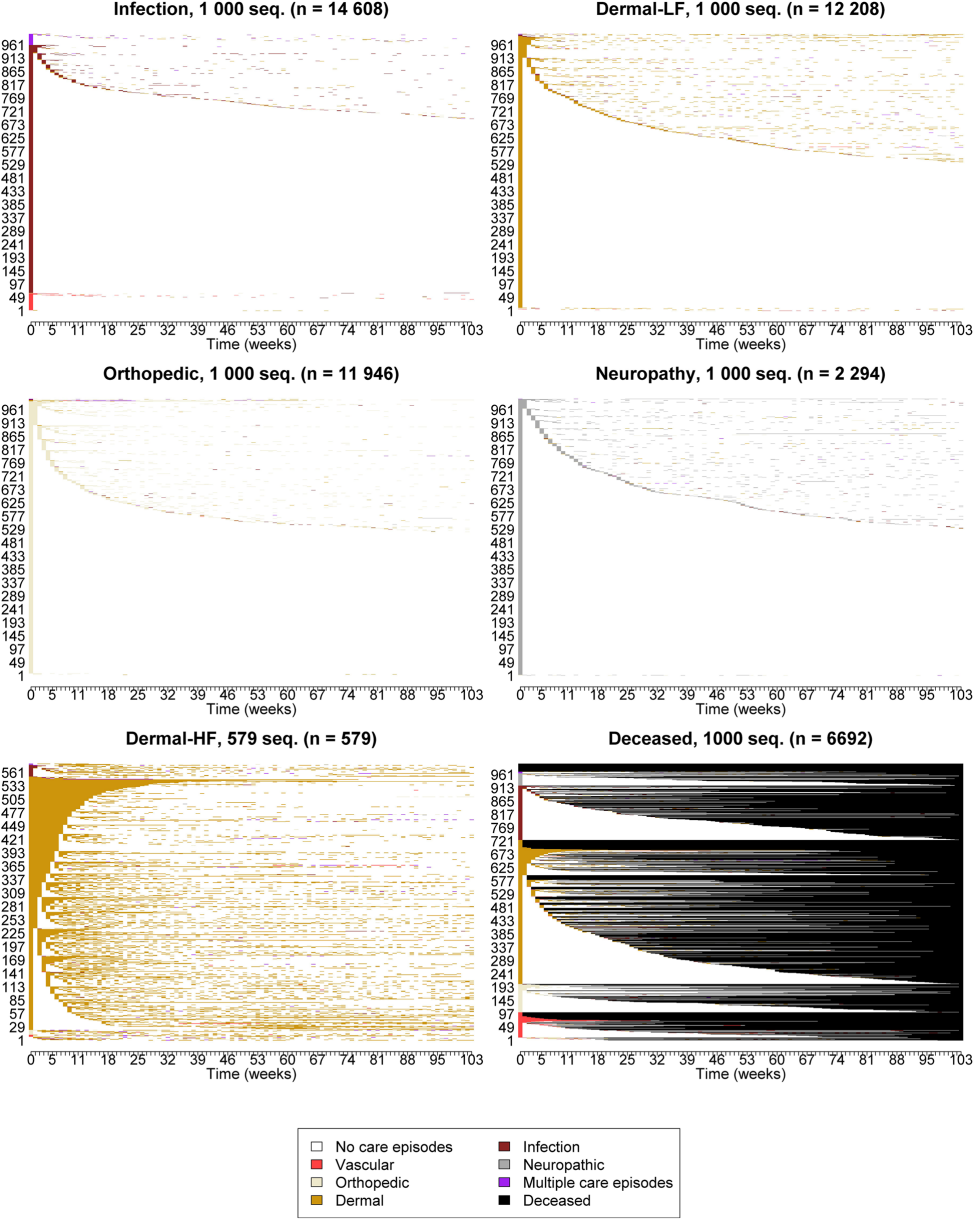
Groups of pathways obtained by sequence analysis. For each group of pathways (except Dermal-HF), a random sample of 1000 pathways is presented. Pathways are sorted by states at the successive positions, starting from the beginning

We initially identified a total of eight groups of pathways, of which four were notably large, and the other four only comprised hundreds of sequences. The pathways in the three smallest groups (*n =* 579, 1–5% of the study population) almost entirely comprised dermal (ulcers, corns and callosities, epidermal thickenings, gangrene, ingrowing nails) care episodes and differed solely in the timing of visits. We interpreted these pathways to represent similar phenomenon and they were regarded as a single group of pathways (Dermal-HF). The smallest group of pathways (*n =* 5) was considered to be an outlier and was excluded from the analysis. Consequently, a total of five groups of pathways were identified together with the group of patients who died during the two-year follow-up (Fig. 1).

Each of the group of pathways mainly included care episodes from a single diagnosis group and was named accordingly: Infection, Dermal-LF (low frequency), Orthopedic, Neuropathy, Dermal-HF (high frequency) and Deceased. The Deceased group, which comprised people who had died during follow-up, was more heterogeneous than the other groups of pathways and included care episodes under all labels. Three groups stood out due to their size: Infection, Orthopedic and Dermal-LF groups comprised 30.2%, 25.2% and 24.7% of all pathways, respectively, while Neuropathy (4.7%), Dermal-HF (1.2%) and Deceased (13.8%) groups were smaller.

In the four largest groups of pathways (Infection, Orthopedic, Dermal-LF and Neuropathy), the majority of pathways (52% to 66% of pathways in the group) comprised only single care episodes. Pathways including few – from two to eight care episodes – were also common (Fig. 1 and Supplementary Fig. 2). Pathways with a higher number of care episodes were rare. The Dermal-HF group made an exception: it had a notably larger variability in the number of care episodes compared to the other groups. A substantially large proportion of its pathways were observed to have a high level of care episodes during the first weeks after entry.

### Background characteristics of patients by group of pathways

On average, patients were over 65 years old and, at the start of the follow-up, had lived with diabetes for over a decade (Table 2). The mean age ranged from 62 to 69 years in groups of pathways other than the Deceased, in which the mean age was over 10 years higher than the overall mean. In all groups of pathways, the average duration of diabetes exceeded 10 years, closely following the patients’ mean age.

**Table 2:** Characteristics of patients by identified groups of pathways

	Infection	Dermal-LF	Orthopedic	Neuropathy	Dermal-HF	Deceased	Total
n (%)	14,608 (30)	12,208 (25)	11,946 (25)	2,294 (5)	579 (1)	6,692 (14)	48,327 (100)
Age, mean (sd)	64 (16)	66 (16)	62 (14)	65 (13)	69 (13)	78 (11)	66 (16)
Years since diabetes diagnosis, mean (sd)	10 (9)	12 (10)	10 (10)	13 (11)	14 (11)	14 (10)	11 (10)
Type 2 diabetes, %	86	85	85	82	85	92	86
Men, %	53	52	40	66	62	54	51
**Comorbid late-stage complications, %**							
Coronary artery disease	2.2	1.1	1.5	5.4	6.2	5.3	2.4
Retinopathy	0.7	0.6	1.2	4.1	4.1	0.4	1.0
Neuropathy	1.1	0.8	1.6	54.2	3.6	2.7	3.9
Nephropathy	0.7	0.5	0.9	4.2	4.1	2.8	1.2

*sd* standard deviation

The majority of patients were diagnosed with type 2 diabetes (Table 2). The distribution of diabetes type differed only slightly between patients in different groups of pathways. Proportion of type 2 diabetes was higher in the Deceased group than patients in the other groups.

Despite being rare in overall, comorbid late-stage complications were more common among patients in the Neuropathy, Dermal-HF and Deceased groups compared to patients in the other groups (Table 2). Coronary artery disease was the most prevalent complication in all groups, with the exception of the Neuropathy group. Naturally, comorbid neuropathy was substantially more common in the Neuropathy group than in any other group*.*

The proportion of males was slightly higher in almost all groups of pathways (Table 2), with the exception of the large Orthopedic group, in which the proportion of females was almost two-thirds.

### Costs of care pathways

**Fig. 2:**
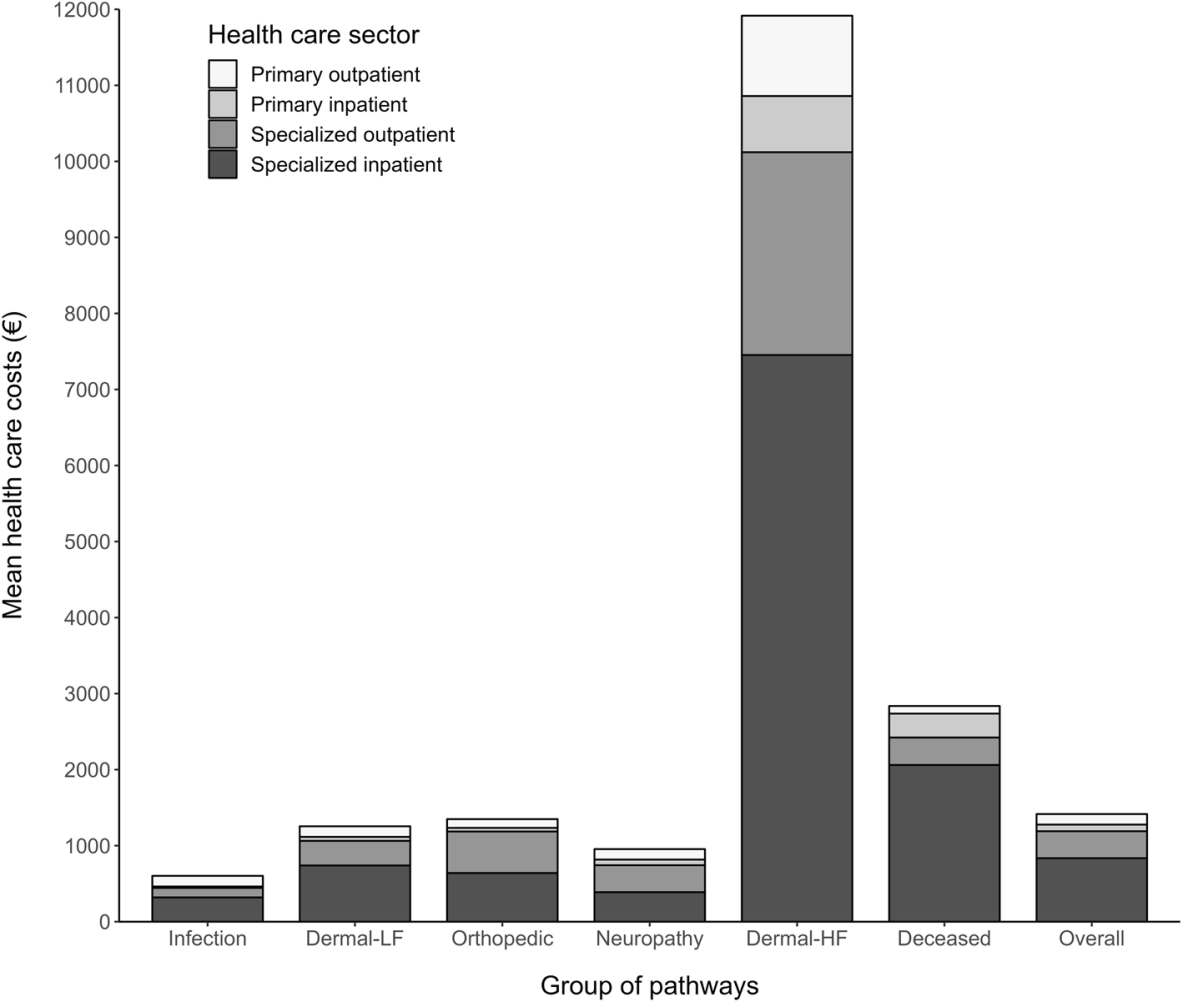
Mean costs of groups of care pathways according to healthcare sector. All costs in 2017 euros

**Fig. 3:**
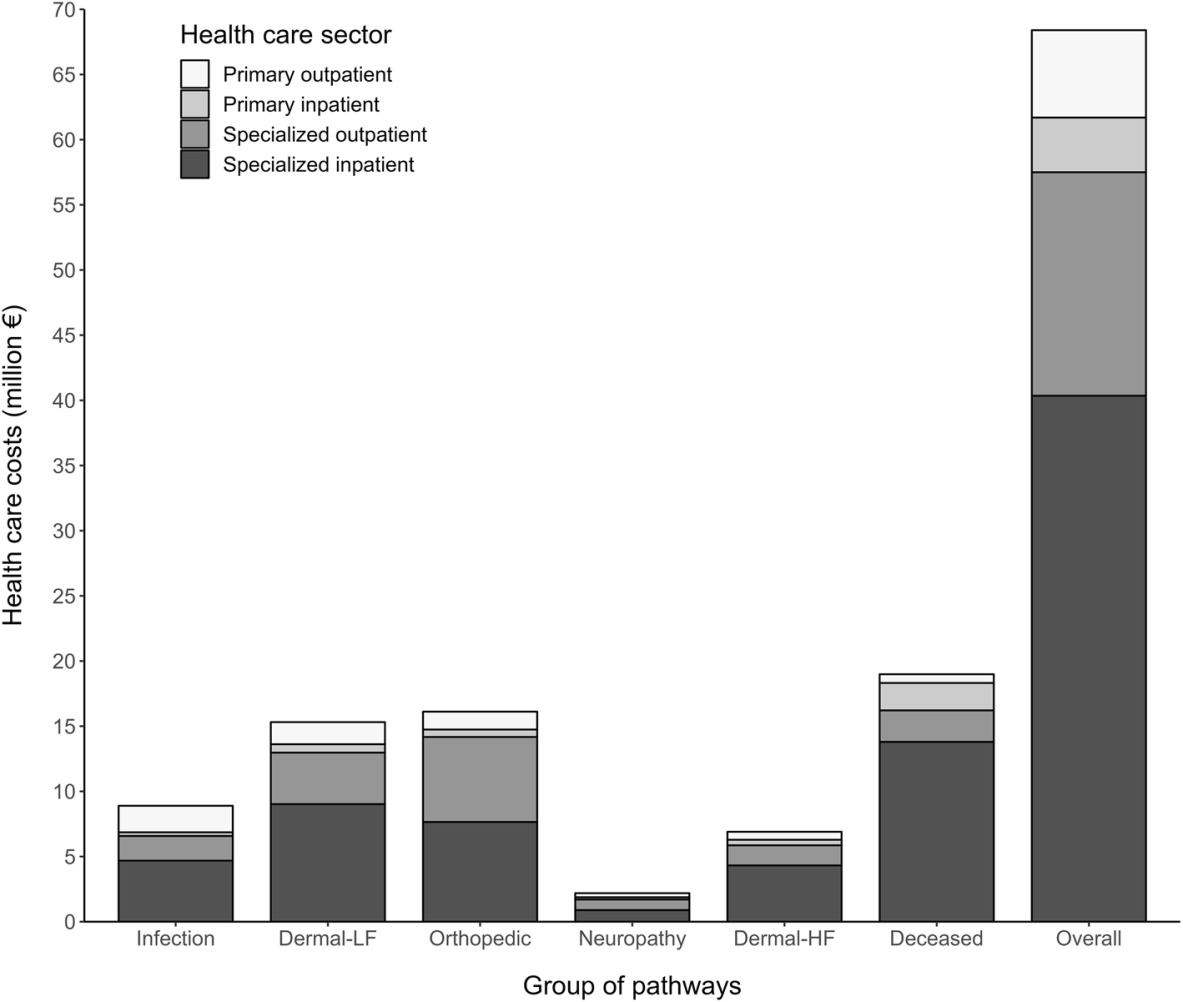
Total costs of groups of care pathways according to healthcare sector. All costs in 2017 euros

The total healthcare costs of care pathways were approximately EUR 70 million (USD 84 million) (Fig. 2, Supplementary Table 1). On average, the pathways cost approximately EUR 1 400 (USD 1680) (Fig. 3, Supplementary Table 1). However, a substantial variability between groups of pathways was observed. The majority of total costs (84% of total costs) resulted from care episodes in specialized health care, while 16% of the total costs were due to primary outpatient and inpatient care. Specialized inpatient care constituted 59% of the total costs.

The Orthopedic group had the highest total costs representing 24% of all costs (Fig. 2, Supplementary Table 1), while the lowest total costs occurred in the Neuropathy group, totaling up to 3% of all costs. The largest (Infection) and smallest (Dermal-HF) group constituted 13% and 10% of total costs, respectively. The mean costs per patient varied considerably between groups of pathways. At highest (Dermal-HF group), the mean costs were over 8-fold higher compared to the mean of the study population while at lowest (Infection group), the mean costs were less than half of the mean of the study population.

The Dermal-HF group markedly differed from the other groups in the number of care episodes and costs (Supplementary Table 1). The number of visits per person to specialized outpatient care was on average 8-fold higher compared to the whole study population. The contribution to the total costs was notable (10%), despite the group’s small size. The mean costs of the Deceased group were over two-fold higher compared to the overall study population, resulting in the highest total costs of all groups. In this group, the costs of specialized inpatient care were over 70%.

In the regression analyses, adjusting for the background variables only slightly modified the differences in mean costs of groups of pathways, with the Neuropathy group as an exception (Supplementary Table 2). The difference of this group to the Infection group vanished after the adjustment. While having comorbid coronary artery disease or nephropathy seemed to substantially increase the costs, diabetes type had no role in the costs given other background variables. We also observed a slight annual increase of mean costs (EUR 75 per year) during the time period.

## Discussion

We identified six distinct groups of pathways, which represented typical care pathways at different stages of the development of foot complications into more severe conditions such as ulcers that could ultimately lead to amputation [1, 23]. We also separately examined the group of patients who had died during the two-year follow-up. Each of the group of pathways mainly comprised care episodes under a single diagnostic category, with the Deceased group as an exception. This reflects the fact that the treatment of foot complications is managed by multiple specialties, each of them responsible for a certain aspect of care or a specific complication [24]. Within the four largest groups (Infection, low frequency Dermal, Orthopedic and Neuropathy), the majority of the complications could be treated at a single treatment episode, after which only a minority of patients required further treatment. In the Deceased group, notably higher level of heterogeneity was observed in the types of care episodes. Nonetheless, dermal care episodes were most prevalent which is consistent with the observation that the risk of mortality within five years increases substantially after initial ulceration [3].

Low and high frequency Dermal groups comprised purely dermal care episodes. These groups represented typical treatment pathways for active ulcers with different levels of need, i.e. high frequency Dermal comprising more severe cases with a need for continuous care. The three other groups (Infection, Orthopedic and Neuropathy) represented care pathways for infections, conditions requiring orthopedic care and neuropathy, which is a major precursor to foot ulcers [1].

Old age and longer duration of diabetes have been associated with elevated risk of foot complications [1]. Our study population had an average age of 66 years and had lived with diabetes for over a decade before the start of their initial care pathway. Overall, the proportions of men and women were approximately equal, however, the sex distributions between groups of pathways differed to some extent. The share of men was higher in the Neuropathy and high frequency Dermal groups which is in line with the observed role of male sex as a risk factor [1]. In addition, other risk factors were more prevalent in the high frequency Dermal group than the other groups: the patients were older and had a longer duration of diabetes, as well as higher prevalence of comorbid late-stage complications. 

The total costs of the studied pathways were approximately EUR 70 million (USD 84 million). Assuming an equal number of pathways per year, this represents an annual cost of EUR 13 million (USD 16 million), which constitutes approximately 1.6% of diabetes-related healthcare costs in Finland (estimates from 2011) [25]. In the UK, the costs of health care for ulceration and amputation were estimated to be around 0.8–0.9% of total NHS budget of which 60% occurred in care episodes in community, outpatient and primary settings [26]. We arrived at a notably lower estimate for the share of total healthcare budget (0.06%), and slightly lower estimate (40%) for the share of primary and outpatient care. However, the estimates are not easily comparable to estimates of annual total costs in the existing literature, due to differences in study design and cost items included.

We observed that the total costs of low and high frequency Dermal groups totaled up to one-third of the total costs for the study population. This differs markedly from the estimate of NHS’s, which states that 90% of foot complication costs were attributable to ulceration [26]. The annual costs of high frequency Dermal group (EUR 5 958) were over eight-fold compared to the overall mean costs and very close to the reported annual cost of ulcer treatment in the UK in 2010–2011 (EUR 5 104) [7].

Even though most of the conditions in the Infection, Orthopedic and Neuropathy groups could be managed with a single treatment episode, their total costs comprised a substantial proportion (40%) of all costs. As treatment options for neuropathy are mainly limited to medication aimed at symptomatic relief [1] and neuropathy suffers from underdiagnosis the total costs remained moderate compared to other groups. The Deceased group had higher total costs compared to other groups of pathways, mainly resulting from large volume of specialized inpatient care. This is not surprising, since healthcare costs have been shown to increase notably in close proximity to death [27].

Costs were to a large extent care-specific: the differences between groups of pathways mainly remained after adjusting for the background variables. The diminished difference between the Neuropathy and Infection groups could be explained by differing patient characteristics: a patient in the Neuropathy group was more likely male and more probably suffered from late-stage complications which both associate with higher costs. Despite differences in the clinical presentation of type 1 and type 2 diabetes [28], we did not observe association between diabetes type and the mean costs. This could reflect the fact that care guidelines do not specify care according to diabetes type [11].

The variability in the number of care episodes and pathway-specific costs is most probably attributable to two main factors: the diversity in the individual need for care and regional differences in the resource availability and organization of care. Multimorbidity is typical for people with diabetic foot, also affecting treatment practice [1]. Accounting for multimorbidity requires tailoring of services to meet the individual needs of patients, possibly increasing the number of care episodes as well as the costs of each care episode [6]. As regular surveys among Finnish diabetes professionals have pointed out, some areas are unable to reach the standards set in the national current care guidelines [12, 29]. However, specific regional estimates are not available.

We limited our perspective to health care and only included the costs of diabetes foot complications. However, individuals and households living with diabetes may also encounter substantial non-healthcare costs, for example, through lost working ability and social care. This generates an additional economic burden on society through lost productivity and subsequent social security costs. A recent study in Finland associated diabetes foot complications with increased risk of early exit from the workforce and productivity loss [30].

Based on reliable and extensive register-based data, we characterized the typical care pathways of patients with diabetic foot using an analysis method that grouped similar care pathways. While we believe that this method is a powerful tool for extracting information from extensive data, it requires subjective decisions regarding its parameters (e.g. time origin and unit, definition of states, measures of similarity). We conducted several alternative analyses with different choices and the results were not particularly sensitive to the chosen parameter values. We aimed at providing an overall picture of the typical care pathways for foot complications and therefore defined the states in our analysis to represent groups of complication diagnoses and excluded examination of procedures. In a study design with focus on a particular endpoint (such as amputation), a more detailed approach would be justified.

Presently, many health care systems throughout the world face the challenge of an aging population and an increasing diabetes prevalence. This places considerable stress on the individual well-being and sustainability of healthcare systems. Finland is not immune to these challenges: the proportion of people aged 65 or over is predicted to increase from 20% to nearly 30% in the next 30 years and the healthcare costs of diabetes are expected to follow a similar trend [31]. The resources available for foot complication treatment are likely to remain scarce in the near future. Thus, better prevention and timely diagnosis to prevent the development of diabetic foot can only be achieved using cost-effective technologies and the more efficient organization of care. Ensuring adequate secondary prevention services and enhancing the patient-centered collaborative care of diabetes should be given a high priority. In the near future, a national register of diabetes care will be launched in Finland [32]. This will enable better monitoring of the quality, effectiveness and costs of diabetes care on a national level.

The main strength of our study is that the analyses are based on population-wide individual-level data. This enables the reliable estimation of service use and costs for people with diabetes foot complications. However, the majority of diagnosing in primary outpatient care is conducted using International Classification of Primary Care 2 (ICPC-2) codes [33]. Due to the inaccuracy of the codes, we could not identify all primary outpatient care episodes related to foot complications. This could result in the underestimation of primary care episodes in the pathways, e.g., the treatment for active ulcers is typically provided by public health nurses after an initial visit to a primary care or specialized care unit and is provided at a patient’s residence, sometimes even on a daily basis. Since these visits are registered as primary care visits using ICPC-2 coding, it is possible that some of the care episodes were excluded from the pathways. We believe this limitation has the largest effect on the pathways of the groups with a high frequency of dermal care episodes.

Information about certain relevant costs such as outpatient medication and diabetic footwear was not included in the data. Thus, our estimates are conservative, which could explain the rather large deviance from the ADA’s and NHS’s estimates of diabetic foot care costs. In Finland, the costs of outpatient medication is shared by the patient and national health insurance and therefore the healthcare system does not directly bear these costs. However, diabetic footwear is provided for patients by the health services and the costs can constitute a notable proportion of the pathway’s overall costs in the shorter pathways.

The reasons underlying the heterogeneity of pathways requires further research. Treatments recommended in the national guidelines are also considered from the perspective of their cost-effectiveness with the aim of improving the provision of health care. Thus, it would be useful to investigate how the national guidelines related to diabetic foot are followed among different specialties and regions and which factors complicate the provision of adequate care. The mapping of regional care practices for diabetes foot complications, together with assessments of their effectiveness, would help identify the best management strategies and enable the co-creation of more effective diabetic foot care.

## Conclusions

Based on reliable and extensive register-based data, we characterized the typical care pathways of patients with diabetic foot using an analysis method that has not been widely applied in health services research previously.

We identified six distinct groups of pathways, each of which mainly comprised care episodes under a single diagnostic category. Majority of pathways comprised only a single care episode, however, among longer pathways we observed notable within and between group variability in the number of care episodes and pathway-specific costs.

Our study contributes by providing a more coherent picture of status quo of diabetes foot complication management in Finland from the viewpoint of health services. The results may aid decision-makers in planning of care pathways and guide further research. Health care professionals may utilize the knowledge of typical care pathways and their patient profiles to detect the patients with high-risk of foot complications at an early stage, to tailor the pathways and to identify appropriate timing for interventions. This could prevent development of more expensive late-stage complications and free resources to other uses. As the prevalence of diabetes is expected to increase in the near future, continuous follow-up of costs and effectiveness of diabetes treatments is crucial in order to meet the growing need.

## Supplementary Information

**Additional file 1. **Flow chart for defining the study population. Number of people excluded in each step is indicated by a dashed box.

**Additional file 2. **Most typical care pathways in each group of pathways. Within groups, pathways are stratified according to type and number of care episodes.

**Additional file 3: ** Costs of care pathways in the study population and by group (all costs in year 2017 euros).

**Additional file 4: ** Regression coefficients (95% confidence interval) from the two models. The intercept term can be interpreted as the mean cost of the Infection group, and the regression coefficients for other groups as differences (95% CI of the difference) to the infection group. Model 1 estimates the crude means and differences, and Model 2 estimates the patient-profile adjusted differences.

## Data Availability

The data that support the findings of this study are available from the Finnish Institute of Health and Welfare but restrictions apply to the availability of these data, which were used under license for the current study, and so are not publicly available. Data are however available from the authors upon reasonable request and with permission of the Finnish Institute of Health and Welfare.

## Abbreviations

ICPC-2: International Classification of Primary Care 2
ADA: American Diabetes Association
FinDM: Diabetes in Finland
DRG: Diagnosis related group
ICD-10: International Classification of Diseases 10
NHS: National Health Service
UK: United Kingdom
