# Supplementary figures and images for: Foot-related diabetes complications: care pathways, patient profiles and costs
Source: BMC Health Serv Res. 2022 Apr 26;22:559. doi: 10.1186/s12913-022-07853-2 (PMC9040351; doi:10.1186/s12913-022-07853-2)

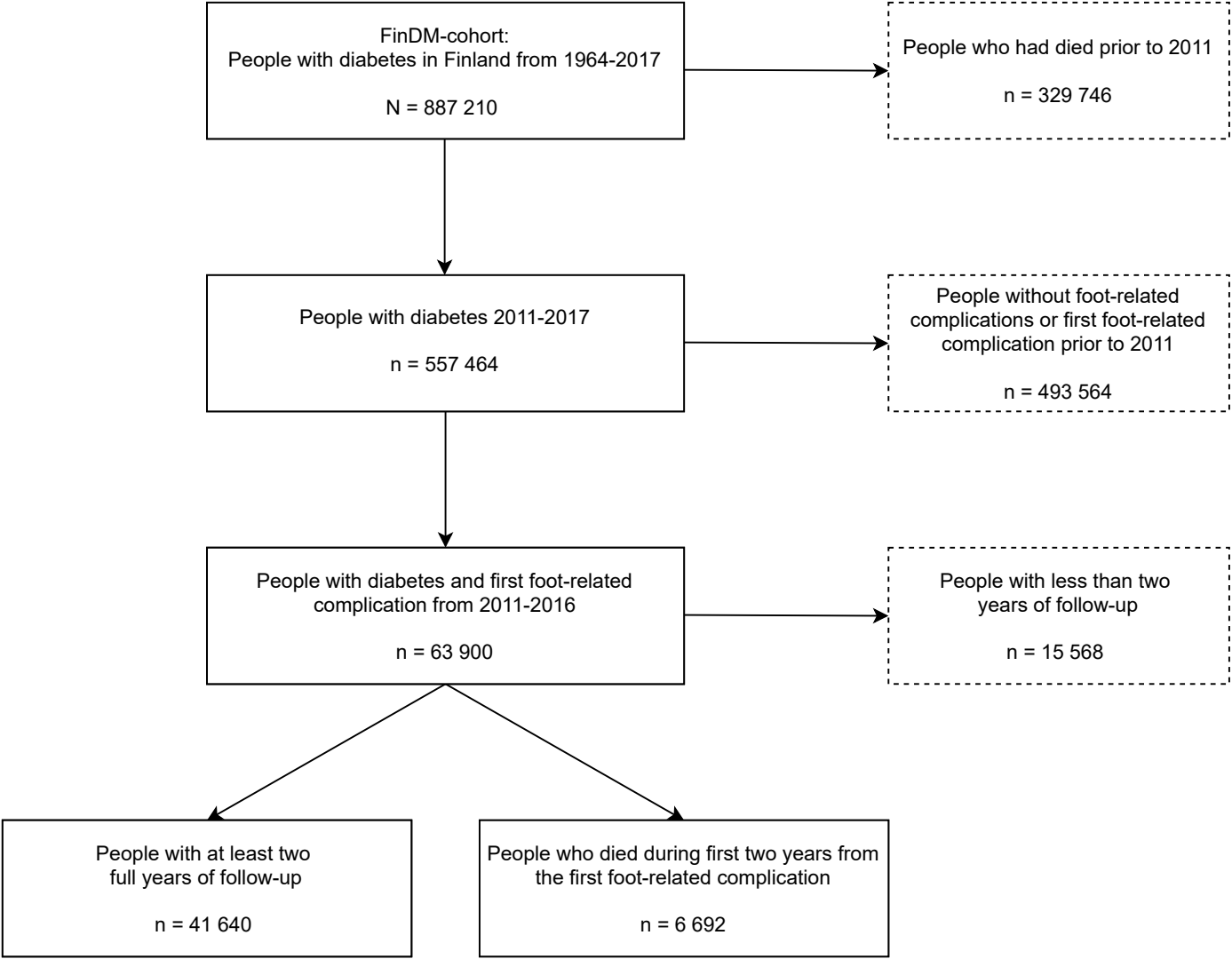

Supplement: Supplementary file 1 — Additional file 1. Flow chart for defining the study population. Number of people excluded in each step is indicated by a dashed box. [file 12913_2022_7853_MOESM1_ESM.pdf]

## Group of pathways 1

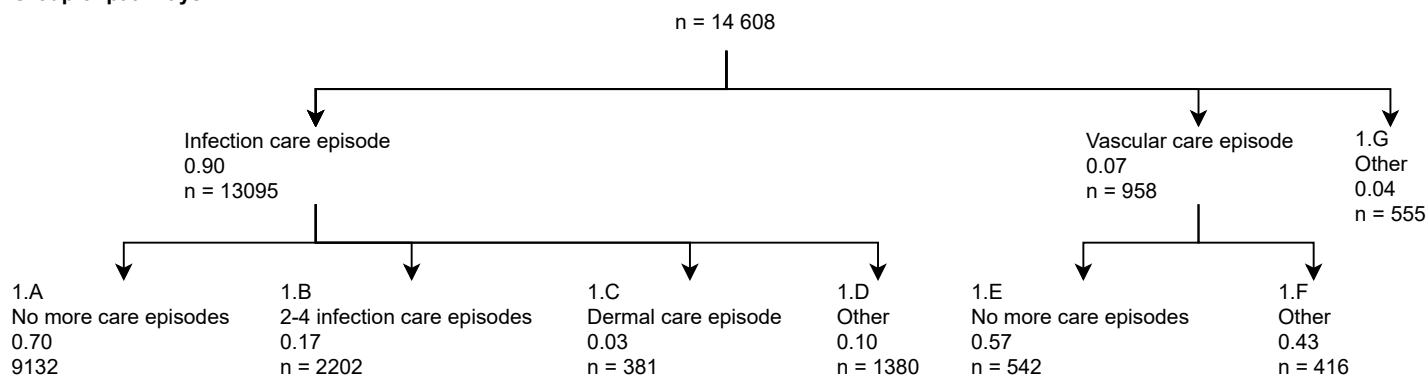

## Group of pathways 2

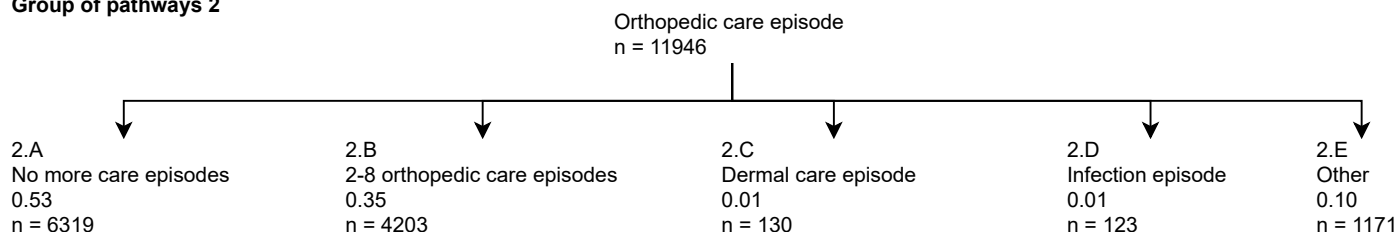

## Group of pathways 3

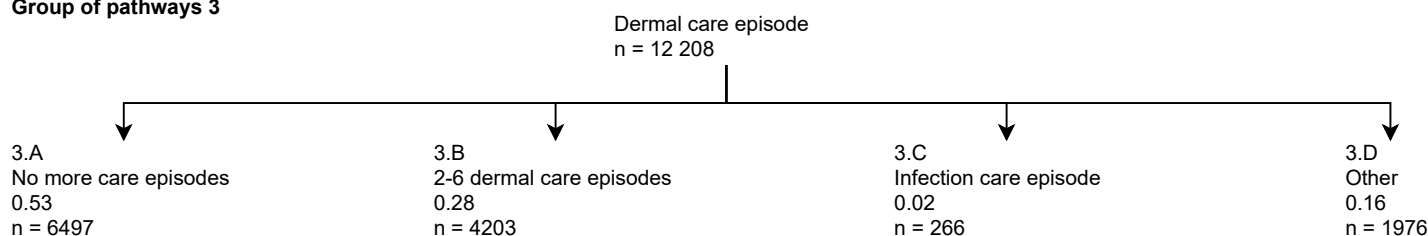

## Group of pathways 4

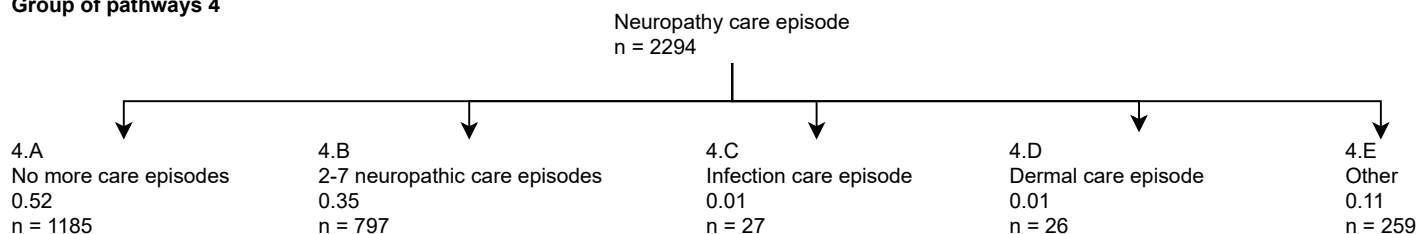

## Group of pathways 5

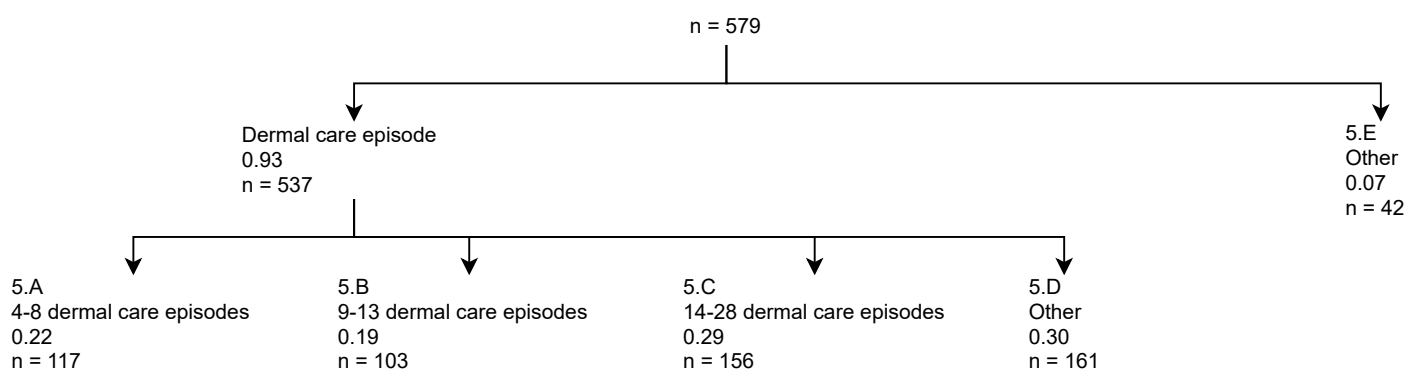

Supplement: Supplementary file 2 — Additional file 2. Most typical care pathways in each group of pathways. Within groups, pathways are stratified according to type and number of care episodes. [file 12913_2022_7853_MOESM2_ESM.pdf]
