# Supplementary material for: Foot-related diabetes complications: care pathways, patient profiles and costs
Source: BMC Health Serv Res. 2022 Apr 26;22:559. doi: 10.1186/s12913-022-07853-2 (PMC9040351; doi:10.1186/s12913-022-07853-2)
Supplement: Supplementary file 3 — Additional file 3: Costs of care pathways in the study population and by group (all costs in year 2017 euros). [file 12913_2022_7853_MOESM3_ESM.docx]

***Supplementary Table 1.*** *Costs of care pathways in the study population and by group (all costs in year 2017 euros).*

|  | Infection | Dermal1 | Orthopedic | Neuropathy | Dermal2 | Deceased | Study population |
| --- | --- | --- | --- | --- | --- | --- | --- |
| n | 14,608 | 12,208 | 11,946 | 2,294 | 579 | 6,692 | 48,327 |
| Primary care |  |  |  |  |  |  |  |
| Outpatient |  |  |  |  |  |  |  |
| Visits, median (IQR) | 1 (1, 1) | 1 (0, 2) | 1 (0, 1) | 1 (0, 1) | 1 (0, 12) | 0 (0, 1) | 1 (0, 1) |
| Visits, total | 19,629 | 21,088 | 13,111 | 3,187 | 9,833 | 7,175 | 74,023 |
| Costs EUR, mean (sd) | 140 (157) | 139 (234) | 115 (178) | 138 (512) | 1,057 (5,001) | 100 (474) | 139 (618) |
| Costs EUR, total | 2,038,350 | 1,697,687 | 1,374,610 | 316,132 | 611,911 | 667,531 | 6,706,219 |
| Inpatient |  |  |  |  |  |  |  |
| Stays, median (IQR) | 0 (0, 0) | 0 (0, 0) | 0 (0, 0) | 0 (0, 0) | 0 (0, 1) | 0 (0, 0) | 0 (0, 0) |
| Stays, total | 518 | 742 | 798 | 145 | 434 | 1,783 | 4,420 |
| Costs EUR, mean (sd) | 19 (228) | 53 (515) | 48 (465) | 74 (1,201) | 739 (1,905) | 315 (1,273) | 87 (698) |
| Costs EUR, total | 278,811 | 641,775 | 570,040 | 170,133 | 428,012 | 2,105,496 | 4,194,268 |
| Specialized care |  |  |  |  |  |  |  |
| Outpatient |  |  |  |  |  |  |  |
| Visits, median (IQR) | 0 (0, 0) | 0 (0, 1) | 0 (0, 2) | 0 (0, 1) | 5 (1, 12) | 0 (0, 1) | 0 (0, 1) |
| Visits, total | 6,778 | 1,3467 | 18,007 | 2,696 | 5,151 | 7,624 | 53,723 |
| Costs EUR, mean (sd) | 130 (526) | 324 (989) | 546 (1,181) | 355 (733) | 2,667 (4,481) | 361 (958) | 355 (1,075) |
| Costs EUR, total | 1,893,337 | 3,955,718 | 6,521,290 | 815,073 | 1,544,391 | 2,417,767 | 17,147,576 |
| Inpatient |  |  |  |  |  |  |  |
| Stays, median (IQR) | 0 (0, 0) | 0 (0, 0) | 0 (0, 0) | 0 (0, 0) | 1 (0, 2) | 0 (0,0) | 0 (0,0) |
| Stays, total | 986 | 1,628 | 2,100 | 224 | 723 | 2,494 | 8,155 |
| Costs EUR, mean (sd) | 321 (2,067) | 739 (3,373) | 640 (2,650) | 388 (1,886) | 7,453 (11,735) | 2,061 (5,408) | 835 (3,549) |
| Costs EUR, total | 4,683,910 | 9,017,661 | 7,649,754 | 888,980 | 4,315,341 | 13,793,726 | 40,349,372 |
| **Total costs EUR, mean (sd)** | 609 (2,359) | 1,254 (4,021) | 1,349 (3,367) | 955 (2,514) | 11,917 (14,838) | 2,837 (6,176) | 1,415 (4,313) |
| **Total costs EUR** | 8,894,408 | 15,312,841 | 16,115,694 | 2,190,318 | 6,899,655 | 18,984,520 | 68,397,436 |

IQR stands for interquartile range, SD stands for standard deviation. Mean and total costs are rounded.
