# Supplementary material for: Foot-related diabetes complications: care pathways, patient profiles and costs
Source: BMC Health Serv Res. 2022 Apr 26;22:559. doi: 10.1186/s12913-022-07853-2 (PMC9040351; doi:10.1186/s12913-022-07853-2)
Supplement: Supplementary file 4 — Additional file 4: Regression coefficients (95% confidence interval) from the two models. The intercept term can be interpreted as the mean cost of the Infection group, and the regression coefficients for other groups as differences (95% CI of the difference) to the infection group. Model 1 estimates the crude means and differences, and Model 2 estimates the patient-profile adjusted differences. [file 12913_2022_7853_MOESM4_ESM.docx]

***Supplementary Table 2.*** *Regression coefficients (95% confidence interval) from the two models. The intercept term can be interpreted as the mean cost of the Infection group, and the regression coefficients for other groups as differences (95% CI of the difference) to the infection group. Model 1 estimates the crude means and differences, and Model 2 estimates the patient-profile adjusted differences.*

|  | Model 1 | Model 2 |
| --- | --- | --- |
| Intercept | 609 (550, 668) | 742 (593, 890) |
| Group of pathway membership* |  |  |
| Low frequency Dermal | 740 (652, 829) | 804 (716, 892) |
| Orthopedic | 645 (558, 733) | 622 (535, 709) |
| Neuropathy | 346 (185, 507) | -2.0 (-162, 158) |
| High frequency Dermal | 11,308 (11,044, 11,611) | 10,914 (10,615, 11,214) |
| Female sex |  | -290 (-360, -219) |
| Age |  | -2.8 (-5.8, 0.3) |
| Age^2 |  | -0.2 (-0.3, -0.1) |
| Diabetes type** |  | -34 (-178, 111) |
| Years with diabetes |  | 24 (19, 28) |
| Pathway start year |  | 76 (51, 100) |
| Comorbid late-stage complications*** |  |  |
| Coronary artery disease |  | 3,109 (3,483, 4,197) |
| Retinopathy |  | 405 (60, 749) |
| Nephropathy |  | 3,840 (2,854, 3,365) |

* Reference: Infection group

** Reference: type 1 diabetes

*** Reference: no comorbid late-stage complication diagnosis
